# The influence of exercise and BMI on injuries and illnesses in overweight and obese individuals: a randomized control trial

**DOI:** 10.1186/1479-5868-7-1

**Published:** 2010-01-06

**Authors:** Carol A Janney, John M Jakicic

**Affiliations:** 1 Western Psychiatric Institute and Clinic, University of Pittsburgh Medical Center, Pittsburgh, PA, USA; 2 Department of Health and Physical Activity, University of Pittsburgh, Pittsburgh, PA, USA

## Abstract

**Background:**

Medically treated injuries have been shown to increase with increasing body mass index (BMI). Information is lacking on the frequency and type of injuries and illnesses among overweight and obese adults who engage in regular physical activities as part of weight loss or weight gain prevention programs.

**Methods:**

Sedentary adults with BMIs between 25 and 40 kg/m^2 ^(n = 397) enrolled in one of two randomized clinical trials that emphasized exercise as part of a weight loss or weight gain prevention program. Interventions differed by duration of the exercise goal (150, 200, or 300 minutes/week or control group). Walking was prescribed as the primary mode of exercise. At six month intervals, participants were asked, "During the past six months, did you have any injury or illness that affected your ability to exercise?" Longitudinal models were used to assess the effects of exercise and BMI on the pattern of injuries/illnesses attributed to exercise over time; censored linear regression was used to identify predictors of time to first injury/illness attributed to exercise.

**Results:**

During the 18-month study, 46% reported at least one injury/illness, and 32% reported at least one injury that was attributed to exercise. Lower-body musculoskeletal injuries (21%) were the most commonly reported injury followed by cold/flu/respiratory infections (18%) and back pain/injury (10%). Knee injuries comprised one-third of the lower-body musculoskeletal injuries. Only 7% of the injuries were attributed to exercise alone, and 59% of the injuries did not involve exercise. BMI (p ≤ 0.01) but not exercise (p ≥ 0.41) was significantly associated with time to first injury and injuries over time. Participants with higher BMIs were injured earlier or had increased odds of injury over time than participants with lower BMIs. Due to the linear dose-response relationship between BMI and injury/illness, any weight loss and reduction in BMI was associated with a decrease risk of injury/illness and delay in time to injury/illness.

**Conclusions:**

Overweight and obese adults who were prescribed exercise as part of weight loss or weight gain prevention intervention were not at increased risk of injury compared to overweight adults randomized not to participate in prescribed exercise. Since onset of injury/illness and pattern of injuries over time in overweight and obese individuals were attributed to BMI, weight reduction may be an avenue to reduce the risk of injury/illness in sedentary and previously sedentary overweight and obese adults.

**Trial Registration:**

Clinicaltrials.gov NCT00177502 and NCT00177476

## Background

Approximately two-thirds of Americans are overweight (BMI 25-29.9 kg/m^2^) or obese (BMI ≥ 30 kg/m^2^)[1]. Recently, medically treated injuries have been shown to increase with increasing BMI[2]. The odds of sustaining a medically treated injury were 15-48% greater among overweight or obese individuals compared to normal weight individuals[2]. Alteration of immune function and an increased risk of infection have also been associated with obesity[3,4]

Physical activity has been promoted as a means to reduce the risk of obesity and of several chronic diseases such as cardiovascular disease, diabetes, depression and certain cancers[5]. The American College of Sports Medicine and the American Heart Association recommend that healthy adults exercise at moderate intensity for at least 30 minutes, 5 days each week or at vigorous intensity for at least 20 minutes, 3 days each week[6]. The primary adverse event of physical activity is injury, and injuries are the primary reason individuals stop exercising[7]. Although limited, several studies suggest that moderate levels of physical activity may reduce the risk and duration of upper respiratory tract infections in adults compared to physically inactive or highly active individuals[3,8-10]. While weight loss has been shown to compromise immune function, moderate exercise as part of a weight loss program may boost the immune system and reduce the incidence and duration of illness as suggested by Scanga and associates[11,12].

Information is lacking on the frequency and type of injuries and illnesses among overweight and obese adults who engage in regular physical activities as part of weight loss or weight gain prevention programs. The purpose of this report was 1) to describe the type and duration of injuries/illnesses sustained, and 2) to determine the independent and combined influences of exercise and BMI on the onset of injury/illness and the pattern of injury/illness over time among overweight and obese participants in a weight loss or weight gain prevention program.

## Methods

### Study Design

Study participants were enrolled in one of two randomized clinical trials (registered with Clinicaltrials.gov; NCT00177502 and NCT00177476) that emphasized exercise as part of a weight loss or weight gain prevention program. At recruitment, participant's BMI was restricted to 25-39.9 kg/m^2 ^in the weight loss study (exercise goal of 200 min/wk) and to 25-29.99 kg/m^2 ^in the weight gain prevention study (control group or exercise goal of 150 or 300 min/wk). Both studies had similar research designs which included 18-month interventions with injuries/illnesses self-reported at 6 month intervals. For the purpose of this analysis and report, the two studies are combined. The study-specific interventions are briefly summarized below.

The weight gain prevention intervention consisted of increasing exercise and modifying eating behaviours. Participants were randomly assigned to gradually progress to 150 or 300 min/wk of moderate-intensity exercise. In addition, participants were instructed to modify eating behaviour in order to emphasize healthier eating patterns, but they were not prescribed an energy restricted diet. Participants received regular in-person or telephone contact throughout the 18 month intervention period to facilitate these changes in eating and exercise behaviours.

The weight loss intervention also consisted of increasing exercise and modifying eating behaviours. However, the exercise goal was to gradually progress to 200 min/wk of moderate-intensity exercise and to reduce energy intake to 1200-1500 kcal/d along with reducing dietary fat intake to 20-30% of total energy intake. Similar to the weight gain prevention study, participants received regular in-person and/or telephone contact throughout the 18 month intervention period to facilitate these changes in eating and exercise behaviours.

Both the weight gain prevention and weight loss studies recommended brisk walking for exercise, five days per week. Participants were permitted to select other activities of moderate intensity based on a perceived exertion of 11 to 13 using the Borg scale[13]. The duration (150, 200 or 300 minutes/week) but not the intensity of the recommended exercise differed among exercise groups. Participants were also instructed to exercise in bouts of at least 10 minutes. Participants recorded their weekly exercise in a log that was returned to the intervention team; feedback was then provided to the participants.

The controls were part of the weight gain prevention study. Participants were provided a self-help manual related to exercise adoption and maintenance, supplemental printed materials related to healthy eating and exercise, and a monthly newsletter. The controls did not receive any additional interventional contact throughout the 18 month period.

### Study Participants

For both studies, participants were excluded for the following reasons: history of myocardial infarction; taking medications that alter heart rate or blood pressure response during exercise (e.g. β-blockers) or that affect metabolism or weight loss (e.g. thyroid medication); treatment for psychological conditions; currently pregnant, pregnant past six months, or planning to become pregnant; medical conditions that could affect metabolism or body weight (e.g. diabetes); reported weight loss > 5% or participated in weight loss or physical activity study during the previous 12 months; or reported exercising regularly for ≥ 20 minutes/day on ≥ 3 days/week over the previous three months. Medications were self-reported by the participants, and no medical tests were performed to assess thyroid status. In addition, the medical screening excluded participants predisposed to exercise related injuries. The medical screening included the following: 1) written medical clearance from a physician verifying that participants had no musculoskeletal condition that would limit or aggravate their ability to engage in moderate intensity physical activity; 2) participants reporting no medical conditions that would limit their ability to engage in moderate intensity exercise; and 3) participants reporting no bone or joint problem (for example, back, knee, or hip) that could be worsened by a change in their physical activity as assessed by the PAR-Q questionnaire[13]. Age was restricted to 18-50 years in the weight loss study and 18-55 years in the weight gain prevention study.

All participants provided written informed consent, and the protocols were approved by the Institutional Review Board of the University of Pittsburgh (Pittsburgh, PA).

### Assessments

#### Injury/Illness

At six month intervals, all participants were asked, "During the past six months, did you have any injury or illness that affected your ability to exercise?" Participants who reported an injury or illness were asked to indicate the type of injuries/illnesses from the following list: heal spur, ankle sprain, shin splints, knee injury, hip injury, back pain/injury, other lower extremity injury (specify), upper body musculoskeletal injury, cold/flu/respiratory illness, allergies, gastrointestinal condition, surgery (specify), or other (specify). Injuries and illnesses were reclassified into broad categories for this report. Participants reported overall how long the injuries/illnesses affected their exercise participation (1 week, 2-4 weeks, 1-2, 2-3, 3-4, 4-5, 5-6 months), how much their exercise was affected (I was unable to exercise, I was able to exercise but had to reduce the amount or intensity, I was able to exercise the usual amount but with some difficulty, my exercise participation was unaffected), and to what extent their injuries/illnesses were caused by their participation in exercise (not at all, a little, moderately, quite a bit, entirely). For the analyses, injuries/illnesses were attributed to exercise if the study participants in either the exercise or control groups reported that exercise caused their injury/illness to any degree (a little, moderately, quite a bit, entirely). These injuries attributed to exercise may have occurred during activities of daily living (walking as part of an errand, shovelling snow, etc.), as part of planned and structured physical activities (bowling, etc.), and for those participants randomized to an exercise group, as part of the prescribed exercise intervention (walking for exercise and other moderate-intensity exercise). Information was not obtained separately for each injury or illness.

#### Exercise

Leisure-time physical activity (kcal/week) was assessed by questionnaire [14]. Energy expenditure was computed using previously published scoring algorithms and classifications based on the compendium of physical activity. This questionnaire queried participants about the amount of walking (12 city blocks equivalent to one mile and one mile equivalent to 20 minutes) and stairs climbed, along with time spent per week in sport, fitness, or recreational activity performed for the purpose of exercise. These data were used to estimate energy expenditure (kilocalories per week) for participation in leisure-time physical activity. Cardiorespiratory fitness was assessed using a submaximal graded exercise test terminated at 85% of age-predicted maximal heart rate.

#### Anthropometric

Body weight was measured using a calibrated balance beam scale with the participant wearing a lightweight hospital gown. Height was measured using a wall-mounted stadiometer with the participant not wearing shoes. BMI was computed as weight/height^2 ^(kg/m^2^). Waist circumference was assessed at the level of the umbilicus, and hip circumference was assessed at the widest point of the buttocks using a Gulick tape measure, with duplicate measures obtained that differed by < 2.0 cm. Waist-to-hip ratio was computed as waist circumference divided by hip circumference.

### Statistical Analysis

Injuries and illnesses were self-reported by participants at six, 12, and 18 months and analyzed with respect to the pattern of injuries/illnesses over time and the time to first injury/illness. Specifically, longitudinal models were used to assess the effects of exercise, BMI, and other covariates on the pattern of injuries/illnesses attributed to exercise over time; censored linear regression [15] was used to identify predictors of time to first injury/illness attributed to exercise. Injuries and illnesses were combined as the single outcome variable since 1) our primary objective was to evaluate the effects of moderate intensity exercise on health and exercise behaviours in overweight and obese adults, and 2) the frequency of exercise attributed injuries or illnesses was insufficient to support separate outcome variables and analyses.

For the censored linear regression models, the data were treated as interval censored since injuries/illnesses were reported for six-month intervals, e.g. an injury/illness reported at six months would have occurred sometime between zero and six months. Models were used to identify potential baseline predictors of 1) time to any injury/illness attributed to exercise and 2) time to lower body musculoskeletal injury attributed to exercise. Baseline risk factors were BMI, leisure-time physical activity, cardiorespiratory fitness, height, waist circumference, waist-to-hip ratio, race, gender, and age. Initially, univariate models were built with the baseline risk factors and prescribed exercise interventions (150, 200, and 300 minutes/week of exercise with the weight gain prevention control group as the reference). As a continuous variable, BMI exhibited a linear relationship with the pattern of injuries/illnesses over time and the time to first injury/illness attributed to exercise in the longitudinal and censored linear regression models. For clinical interpretation and application purposes, this report also modelled BMI as a categorical variable based on widely accepted and established categories of overweight and obesity. Variables were selected for the multivariate model if the p-value ≤ 0.20. With the multivariate model, backwards elimination was performed to obtain a parsimonious model with significant main effects.

Longitudinal analyses were performed to examine the role of exercise and BMI as time-varying covariates on injuries attributed to exercise over time. Separate models were built for any injury/illness and for lower body musculoskeletal injuries attributed to exercise. The outcome was determined for each participant at three possible time points (six, 12, and 18 months) and indicated if injuries or illnesses attributed to exercise occurred during the preceding six months (0 = no, 1 = yes). The population averaged models were fit with generalized estimating equations using an unstructured correlation matrix and robust variance estimator [16]. Missing observations were assumed to be missing at random. One-time covariates defined at baseline were prescribed exercise intervention, age, gender, and race. Time varying covariates assessed at six, 12 and 18 months were BMI, leisure-time physical activity (< 950 as the reference group, 950-1949, 1950-2189, 2190+ kcal/week), and cardiorespiratory fitness (< 620 as the reference group, 620-779, 780-959, 960+ seconds). Initially, univariate models with months were built with the time-varying and one-time covariates. Next, the interaction term of months and the covariate were added to the univariate models. Finally, the association of exercise and BMI were explored as 1) main effects in the same model and 2) an interaction term. A significance level of p < 0.10 was necessary to retain a covariate or interaction term in the multivariate model.

The 2 × k contingency tables with Chi-Square statistics or Fisher's Exact Test were used to compare the frequency of injuries/illnesses and the effect of injuries/illnesses on exercise participation in the control and exercise groups. All analyses were performed on SAS (version 9.1; SAS Institute Inc, Cary, NC) or Stata (version 9.2; StataCorp, College Station, TX).

## Results

As expected, the baseline anthropometric measurements for weight gain prevention participants were statistically smaller than those for weight loss participants (Table 1) due to differences in the BMI enrollment criteria. The weight loss study enrolled more men and African Americans than the weight gain prevention study. Approximately 4%, 12%, and 18% of the participants in the weight gain prevention study were lost to follow-up at six, 12 and 18 months, respectively (p > 0.05 control and exercise groups, data not shown). Greater loss to follow-up was observed among participants in the weight loss study at 12 (31%) and 18 (41%) months but not at six (2%) months compared to participants in the weight gain prevention study.

**Table 1:** Baseline characteristics of adults (n = 397) in behavioral weight loss or maintenance programs (mean ± standard deviations, or n(%)).

Variable	Control Group (n = 77)	Exercise 150 min/wk (n = 64)	Exercise 200 min/wk (n = 172)	Exercise 300 min/wk (n = 84)
Age (years)	44.4 ± 8.0	44.2 ± 8.4	44.0 ± 8.3	45.3 ± 8.3

Height (cm)	165.2 ± 6.7	165.3 ± 6.8	167.7 ± 9.0	166.0 ± 7.6

Weight (kg) ** ^a^	74.2 ± 8.1	74.6 ± 8.2	92.7 ± 14.3	74.2 ± 7.9

BMI (kg/m2) ** ^a^	27.1 ± 1.6	27.3 ± 1.8	32.9 ± 3.4	26.9 ± 1.6

Waist circumference (cm) **^a^	89.9 ± 8.8	90.7 ± 7.9	106.8 ± 11.4	90.2 ± 8.3

Hip circumference (cm) ** ^a^	106.7 ± 5.7	106.0 ± 5.8	116.4 ± 8.1	106.2 ± 5.0

Waist-to-hip ratio **^a^	0.84 ± 0.08	0.86 ± 0.07	0.92 ± 0.08	0.85 ± 0.07

Cardiorespiratory Fitness (minutes to 85% maximal heart rate)	10.3 ± 3.3	10.2 ± 3.7	9.2 ± 4.4	10.4 ± 3.8

Leisure-time physical activity (kcal/week)	944 ± 996	834 ± 728	691 ± 735	692 ± 665

Gender ** ^a^				
Female	70 (91%)	59 (92%)	134 (78%)	77 (92%)
Male	7 (9%)	5 (8%)	38 (22%)	7 (8%)

Race ** ^a^				
White	58 (75%)	50 (79%)	117 (68%)	67 (80%)
Black	13 (17%)	8 (13%)	50 (29%)	13 (15%)
Other	6 (8%)	5 (8%)	5 (3%)	4 (5%)
Missing	0 (0%)	1 (< 1%)	0 (0%)	0 (0%)

^a ^Overweight treatment groups (control group, exercise 150 min/wk, exercise 300 min/wk) enrolled in the weight gain prevention study were significantly different from overweight/obese treatment group (exercise 200 min/wk) enrolled in the weight loss study at p < 0.01(**) or p < 0.05(*) due to recruitment of slightly different study populations for the two randomized clinical trials.

The exercise intervention group compared to the sedentary control group exhibited significantly greater reductions in BMI at 6 (-2.1 ± 2.2 versus -0.4 ± 1.1 kg/m^2^), 12 (-1.9 ± 2.7 versus 0.0 ± 1.7 kg/m^2^), and 18 (-1.7 ± 2.7 versus -0.2 ± 1.4 kg/m^2^) months, respectively (p < 0.05). During the study, approximately one quarter of the study participants achieved BMI < 25 kg/m^2 ^as summarized in Table 2 and reported elsewhere (Jakicic JM, Otto AD, Semler L, Winters C, Polzien K, Lang W, and Mohr K. Effect of exercise on 18-month weight change in overweight adults, submitted). Median leisure-time physical activity for all participants was 524 kcal/week (25^th ^and 75^th ^percentile: 224, 1108 kcal/week) at baseline. From baseline to month 18, the exercise intervention group compared to the sedentary control group exhibited significantly greater changes in leisure-time physical activity (840 kcal/day for the exercise group versus 432 kcal/day for control group, p = 0.03) and cardiorespiratory fitness (3.7 minutes for the exercise groups versus 2.5 minutes for control group, p = 0.06) (data not shown).

**Table 2:** Frequency (%) of participants by BMI (kg/m^2^) categories and prescribed exercise interventions at baseline, 6, 12 and 18 months.

	Prescribed Intervention				
**BMI (kg/m**^2^)	**Control Group** **(n = 77)**	**Exercise 150 min/wk** **(n = 64)**	**Exercise 200 min/wk** **(n = 172)**	**Exercise 300 min/wk** **(n = 84)**	**Total Sample** **(n = 397)**

Baseline					
< 25	0 (0)	0 (0)	0 (0)	0 (0)	0 (0)
25-29.9	77 (100)	64 (100)	84 (100)	32 (19)	251 (63)
30+	0 (0)	0 (0)	0 (0)	140 (81)	146 (37)

6 months					
< 25	16 (23)	14 (23)	21 (12)	18 (22)	69 (18)
25-29.9	53 (75)	44 (71)	79 (46)	63 (75)	238 (62)
30+	2 (3)	4 (6)	69 (41)	2 (2)	77 (20)

12 months					
< 25	18 (27)	12 (23)	20 (16)	19 (25)	69 (21)
25-29.9	44 (66)	35 (66)	51 (40)	55 (71)	185 (57)
30+	5 (7)	6 (11)	57 (45)	3 (4)	71 (22)

18 months					
< 25	12 (22)	13 (25)	17 (17)	25 (36)	67 (24)
25-29.9	40 (74)	35 (67)	33 (33)	40 (58)	148 (54)
30+	2 (4)	4 (8)	52 (51)	4 (6)	62 (22)

Throughout the 18-month study, 46% of all participants reported at least one injury or illness, and 32% reported at least one injury that was attributed to exercise (data not shown). Lower-body musculoskeletal injuries (21%) were the most commonly reported injuries followed by cold/flu/respiratory infections (18%) and back pain/injury (10%) (Table 3). Almost one-third of the lower-body musculoskeletal injuries were attributed to knee injuries. Other lower extremity musculoskeletal injuries that accounted for more than 5% of these injuries included sprains (14%), tendonitis (12%), hip injuries (8%), and general muscular injuries (8%) (data not shown). Only 7% of the injuries were entirely attributed to exercise, and 59% of the injuries did not involve any exercise (Table 3). For 40% of the injuries, participants were unable to exercise due to the injury or illness. For the majority of the injuries (63%), exercise participation was affected for one month or less. Fewer than 14% of the injuries affected exercise participation for three months or longer. Overall, the injury rate averaged 1.8%/month. A higher injury rate (2.7%/month) was observed for the first six months. The distributions of self-reported injuries/illnesses that affected the participant's ability to exercise are summarized in Table 3 for all injuries and illnesses regardless of the reason, and Table 4 for only those injuries and illnesses that were attributed to exercise to any degree (a little, moderately, quite a bit, or entirely) by the participant.

**Table 3:** Distribution of all self-reported injuries/illnesses^a ^that affected the participant's ability to exercise during the studies.

	All self-reported injuries and illnesses^a^			
	
	Number of individuals (%)			Reports (%)
	
Months	0-6 (n = 384)	6-12 (n = 325)	12-18 (n = 277)	0-18 (n = 986)
Injury/Illness				
Lower body musculoskeletal	71 (18)	72 (22)	62 (22)	205 (21)
	
Cold/Flu/Respiratory	67 (17)	66 (20)	40 (14)	173 (18)
	
Back pain/injury	32 (8)	39 (12)	24 (9)	95 (10)
	
Allergies	13 (3)	15 (5)	9 (3)	37 (4)
	
Surgery	18 (5)	11 (3)	7 (3)	36 (4)
	
Upper body musculoskeletal	13 (3)	8 (2)	11 (4)	32 (3)
	
GI Condition	13 (3)	7 (2)	9 (4)	29 (3)
	
Other ^b^	12 (3)	18 (6)	15 (5)	57 (6)

Any injury/illness	175 ^c ^(46)	151 ^c ^(46)	123 ^c ^(44)	449 ^c ^(46)

How long injury/illness affected exercise participation?				
	
1 week	63(36)	34(23)	17(14)	114 (25)
	
2-4 weeks	62(35)	58(38)	48(39)	168 (37)
	
1-2 months	31(18)	21(14)	15(12)	67 (15)
	
2-3 months	7(4)	18(12)	11(9)	36 (8)
	
3-4 months	5(3)	5(3)	11(9)	21 (5)
	
4-5 months	1(< 1)	4(3)	5(4)	10 (2)
	
5-6 months	4(2)	10(7)	15(12)	29 (6)
	
Missing	2(< 1)	1(< 1)	1(< 1)	4(< 1/)

How much was your exercise affected?				
I was unable to exercise	64(37)	69(46)	47(38)	180 (40)
	
I was able to exercise but had to reduce the amount or intensity	92(53)	75(50)	71(58)	238 (53)
	
I was able to exercise the usual amount but with some difficulty	12(7)	6(4)	4(3)	22 (5)
	
My exercise participation was unaffected	7(4)	1(< 1)	1(< 1)	9 (2)

To what extent was this injury/illness caused by your participation in exercise?				
Not at all	115(66)	81(54)	70(57)	266 (59)
	
A little	26(15)	30(20)	25(20)	81 (18)
	
Moderately	16(9)	10(7)	9(7)	35 (8)
	
Quite a bit	14(8)	13(9)	9(7)	36 (8)
	
Entirely	4(2)	17(11)	10(8)	31 (7)

^a ^Participants could report more then one injury or illness Participants may or may not have attributed these injuries and illnesses to exercise.

^b ^Injuries/illnesses that afflicted less than 1% of the study population included cardiovascular, headaches, arthritis, mental health conditions, dental, endocrinology, infections, sore muscles, asthma, anemia, and cancer.

^c ^Denominators for the subsequent percentages in this column

**Table 4:** Distribution of self-reported injuries/illnesses that the participant attributed to exercise and affected their ability to exercise during the studies.

	**Self-reported injuries and illnesses that were attributed to exercise by the participant**^a^			
	**Number of individuals (%)**			**Reports (%)**

**Months**	**0-6** **(n = 384)**	**6-12** **(n = 325)**	**12-18** **(n = 277)**	**0-18** **(n = 986)**

Injury/Illness				
	
Lower body musculoskeletal	43 (11)	47 (14)	39 (14)	129 (13)
	
Cold/Flu/Respiratory	13 (3)	24 (7)	11 (4)	48 (5)
	
Back pain/injury	9 (2)	16 (5)	12 (4)	37 (4)
	
Allergies	7 (2)	11 (3)	2 (< 1)	20 (2)
	
Surgery	3 (< 1)	5 (2)	1 (< 1)	9 (< 1)
	
Upper body musculoskeletal	5 (1)	5 (2)	4 (1)	14 (1)
	
GI Condition	4 (1)	2 (< 1)	3 (1)	9 (< 1)
	
Other ^c^	4 (1)	7 (2)	2 (< 1)	13 (1)

Any injury/illness attributed to exercise	60 ^d ^(16)	70 ^d ^(22)	53 ^d ^(19)	183 ^d ^(19)

How long injury/illness affected exercise participation?				
	
1 week	20(33)	12(17)	5(9)	37 (20)
	
2-4 weeks	17(28)	24(34)	24(45)	65 (36)
	
1-2 months	14(23)	12(17)	5(9)	31 (17)
	
2-3 months	3(5)	9(13)	7(13)	19 (10)
	
3-4 months	3(5)	4(6)	3(6)	10 (5)
	
4-5 months	0(0)	2(3)	3(6)	5 (3)
	
5-6 months	2(3)	7(10)	6(11)	15 (8)
	
Missing	1(< 1)	0(0)	0(0)	1(< 1)

How much was your exercise affected?				
	
I was unable to exercise	21(35)	30(43)	14(26)	65 (36)
	
I was able to exercise but had to reduce the amount or intensity	32(53)	37(53)	37(70)	106 (58)
	
I was able to exercise the usual amount but with some difficulty	6(10)	3(4)	2(4)	11 (6)
	
My exercise participation was unaffected	1(2)	0(0)	0(0)	1 (< 1)

To what extent was this injury/illness caused by your participation in exercise?				
	
Not at all	0 (0)	0 (0)	0 (0)	0 (0)
	
A little	26(43)	30(43)	25(47)	81 (44)
	
Moderately	16(27)	10(14)	9(17)	35 (19)
	
Quite a bit	14(23)	13(19)	9(17)	36 (20)
	
Entirely	4(7)	17(24)	10(19)	31 (17)

^a ^Participants could report more then one injury or illness. Only injuries and illnesses that the participant attributed to exercise (little, moderately, quite a bit, or entirely) are reported. Excluded from the table are injuries and illnesses that the participant's indicated were "not at all" caused by their participation in exercise.

^c ^Injuries/illnesses that afflicted less than 1% of the study population included cardiovascular, headaches, arthritis, mental health conditions, dental, endocrinology, infections, sore muscles, asthma, anemia, and cancer.

^d ^Denominators for the subsequent percentages in this column

The percentage of participants who reported at least one injury/illness or one injury/illness attributed to exercise did not differ between the exercise and control groups (p > 0.44). During the 18 months, the exercise and control groups did not differ in frequency of the seven most commonly reported injuries (lower body musculoskeletal, cold/flu/respiratory, back pain/injury, allergies, surgery, upper body musculoskeletal, and GI condition) for all injuries/illnesses or injuries/illnesses attributed to exercise (p > 0.16)(data not shown). On average, the negative impact of any injury/illness on exercise participation was shorter among participants in the exercise as compared to the control group (p ≤ 0.01): 28% versus 16% were affected for 1 week, 39% versus 34% were affected 2-4 weeks, 12% versus 29% were affected 1-2 months, and 21% versus 22% were affected 2 months or longer for the exercise and control groups, respectively. This association was also observed for injuries and illnesses attributed to exercise (p ≤ 0.01); 22% versus 10% were affected for 1 week, 38% versus 24% were affected 2-4 weeks, 13% versus 38% were affected 1-2 months, and 27% versus 28% were affected 2 months or longer for the exercise and control groups, respectively. Exercise participation due to any injury or illness did not differ between the exercise and control groups (p ≥ 0.12); approximately 40% of those injured in the exercise and control groups were unable to exercise, and 54% of the exercise group and 47% of the control group reported reducing the amount or intensity of their exercise due to the injury or illness. Similar results were observed between the exercise and control groups for exercise participation due to injuries and illnesses attributed to exercise (data not shown). Also, the exercise and control groups reported no differences in the extent to which any injury/illness, or injury/illness attributed to exercise, was caused by exercise participation (p > 0.47) (data not shown).

### Interval Censored Linear Regression

Based on interval censored linear regression models, exercise (defined as prescribed exercise based on randomized intervention assignment, baseline cardiorespiratory fitness, or baseline leisure-time physical activity) was not associated with time to first injury/illness (p ≥ 0.41) or time to first lower body musculoskeletal injury (p ≥ 0.22) attributed to exercise (Table 5 and data not shown). Although not statistically significant, the beta coefficients suggested a weak dose-response relationship between duration of exercise and time to first injury. First, participants prescribed 200-300 min/week of exercise experienced injuries slightly earlier than the control group. Second, overweight adults prescribed 150 min/week of exercise delayed injuries/illnesses attributed to exercise as compared to the control group (Table 5).

**Table 5:** Beta coefficients and standard errors for time to first 1) injury/illness or 2) lower body musculoskeletal injury.

	Self-reported Outcome (time in months)			
	
Independent variables in censored linear regression models	Any injury/illness attributed to exercise		Lower body musculoskeletal injury attributed to exercise	
	
	β ± SE	p-value	β ± SE	p-value
Exercise Intervention ^a^		0.41		0.51
Control	Reference		Reference	
150 minutes/week	0.9 (2.5)		0.4 (2.9)	
200 minutes/week	-2.3 (2.0)		-2.6 (2.3)	
300 minutes/week	-1.8 (2.3)		-2.3 (2.6)	

BMI (kg/m^2^) at baseline ^b, c^	-0.51 (0.18)	0.005	-0.39 (0.21)	0.06

^a ^The β (SE) for the exercise (150, 200 or 300 minutes/week) versus the control group was -1.5 (1.9), p = 0.41 for any injury/illness attributed to exercise and -1.9 (2.2), p = 0.37 for lower body musculoskeletal injury attributed to exercise.

^b ^The β (SE) for obese (≥ 30 kg/m^2^) versus overweight (25-29.9 kg/m^2^) participants at baseline was -2.6 (1.5), p = 0.08 for any injury/illness attributed to exercise and -2.4 (1.7), p = 0.16 for lower body musculoskeletal injury attributed to exercise.

^c ^centered at 30

Since no association was observed between the randomized exercise prescription, baseline cardiorespiratory fitness or baseline leisure-time physical activity and time to first injury, all participants in the exercise and control groups were used to study the role of BMI on time to first injury attributed to exercise. BMI at baseline was a significant predictor of time to first injury/illness and time to first lower body musculoskeletal injury attributed to exercise (Table 5). Participants with higher baseline BMIs were injured earlier than participants with lower baseline BMIs (injuries occurred approximately two weeks earlier for each unit increase in BMI). On average, obese participants were injured 2.6 ± 1.5 months earlier than overweight participants for any injury or illness attributed to exercise. Similar results were obtained when the analyses were restricted to only participants randomized to an exercise program (data not shown). This association between BMI and injuries remained after controlling for prescribed exercise intervention and exercise duration, suggesting that baseline BMI was a significant contributor to the onset of injury/illness regardless of whether or not participants were prescribed exercise as part of the intervention. Age, gender, race, waist circumference, and waist-to-hip ratio at baseline were not predictive of time to first injury/illness or time to first lower musculoskeletal injury attributed to exercise (p > 0.19).

### Longitudinal Analyses

No longitudinal association was observed between any injury/illness or lower body musculoskeletal injury and exercise regardless of the definition of exercise (prescribed intervention, cardiorespiratory fitness, or leisure-time physical activity) (p ≥ 0.19, Table 6 and data not shown). Also, no longitudinal association was observed between exercise duration and any injury or lower body musculoskeletal injury attributed to exercise when the analyses were restricted to overweight participants only (p ≥ 0.32, data not shown). However, the risk of injury attributed to exercise was slightly elevated for overweight participants randomized to 300 min/wk of exercise compared to sedentary controls (OR [95% CI] = 1.55 [0.85, 2.81] for any injury/illness and 1.41 [0.70, 2.83] for lower body musculoskeletal injury attributed to exercise).

**Table 6:** Odds ratio (95%CI) for either any injury/illness or lower body musculoskeletal injury attributed to exercise.

Independent variable in longitudinal model	Self-reported injury or illness attributed to exercise (0 = no, 1 = yes) at months 6, 12, and 18			
	
	Any		Lower body musculoskeletal	
	
	Odds ratio (95%CI)	p-value	Odds ratio (95%CI)	p-value
Exercise Intervention ^a, b^		0.59		0.74
Control	Reference		Reference	
150 minutes/week	1.09 (0.54,2.20)		1.00 (0.45,2.22)	
200 minutes/week	1.18 (0.66,2.11)		1.32 (0.69, 2.52)	
300 minutes/week	1.50 (0.81, 2.78)		1.31 (0.64, 2.67)	

BMI (kg/m^2^) ^c, d, e^	1.10 (1.05,1.16)	< 0.001	1.05 (0.99, 1.12)	0.09

Race ^a^		0.007		0.01
White	Reference		Reference	
Black	0.44 (0.25,0.75)		0.46 (0.13, 1.56)	
Other	0.56 (0.21,1.50)		0.44 (0.24, 0.80)	

^a ^one-time covariate defined at baseline

^b ^The OR (95% CI) for the exercise intervention (150, 200 or 300 minutes/week) versus the control group was 1.26 (0.74, 2.13), p = 0.40 for any injury/illness attributed to exercise and 1.24 (0.68, 2.25), p = 0.49 for lower body musculoskeletal injury attributed to exercise.

^c ^time-varying covariate defined at months 6, 12, and 18

^d ^centered at 30

^e ^Controlling for race and randomized exercise intervention, the odds of injury (95% CI) over time for overweight (BMI 25-29.9 kg/m^2^) and obese (BMI > 30 kg/m^2^) participants were 1.65 (1.00, 2.70) and 1.89 (1.06, 3.33) for any injury and 1.53 (0.89, 2.63) and 1.28 (0.67, 2.47) for lower body musculoskeletal injury attributed to exercise, respectively, compared to participants who achieved BMIs < 25 kg/m^2^.

BMI was significantly associated with any injury/illness and lower body musculoskeletal injury attributed to exercise over time. The odds of any injury attributed to exercise increased 6-10% for each unit increase in BMI regardless of whether or not the participant was randomized to the exercise intervention or control groups. Similar results for BMI were obtained when analyses were restricted to only 1) participants randomized to an exercise program (OR [95%CI] = 1.10 [1.04, 1.16] for any injury/illness and 1.05 [0.98, 1.12] for lower body musculoskeletal injuries attributed to exercise), and 2) obese participants randomized to 200 minutes/week at baseline (OR [95%CI] = 1.09 [1.03, 1.15] for any injury/illness and OR [95%CI] = 1.03 [0.97, 1.10]) for lower body musculoskeletal injury attributed to exercise.

The pattern of any injury/illness or lower body musculoskeletal injury attributed to exercise did not vary by time (p ≥ 0.13), gender (p ≥ 0.13), or age (p ≥ 0.85). The odds of any injury/illness or lower body musculoskeletal injury tended to be lower for African Americans and other races than for whites (p = 0.09). None of the interaction terms (months × covariate or exercise × BMI) were significant. In the final multivariate models, BMI and race remained significant predictors of any injury/illness and lower body musculoskeletal injuries attributed to exercise over time (Table 6). Overall, these findings suggested that lowering BMI may result in a corresponding reduction over time in the occurrence of injuries attributed to exercise in overweight and obese adults.

## Discussion

In this clinical trial of overweight and obese adults (baseline BMI between 25 and 39.9 kg/m^2^), being randomized to an exercise program that emphasized walking posed no greater risk of injury/illness than being randomized to the control condition. These results indicate that sedentary overweight and obese adults within the age and BMI ranges of the study participants can be advised to initiate and maintain a moderate-intensity exercise program as part of their weight loss and health promotion efforts. This finding is important in that exercise has been shown to enhance long-term weight loss maintenance [17,18] and to reduce the risk of numerous chronic diseases independent of body weight [5].

Our findings are supported by the Lifestyle Interventions and Independence for Elders Pilot Study[19], in which older adults with an average BMI of 30 kg/m^2 ^were randomized into either a physical activity intervention that emphasized walking or a health education intervention. After one year, there was no difference in the number of participants experiencing a non-serious or serious adverse event between the two interventions. Another study reported few adverse events in a home-based walking program among high-risk cardiovascular male veterans with BMI > 28 kg/m^2 ^[20]. Only one study has reported significantly more injuries in a high (exercise goal of 2500 kcal/wk) versus moderate (exercise goal of 1000 kcal/wk) physical activity intervention for overweight and obese adults in a weight loss study [21]. The higher injury rate may be explained by the fact that the high physical activity group reported significantly greater heavy-intensity activities than the moderate physical activity group[21]. Overall, these studies suggest that overweight and obese adults who walk or exercise at moderate intensity have no greater risk of injury than sedentary overweight or obese adults.

Indicating that body size rather than exercise per se had the greatest influence on both the onset and the pattern of injuries, the present study, to our knowledge, provides the first evidence that overweight and obese individuals can lower their ongoing risk of injury by reducing BMI. Due to the linear dose-response relationship between BMI and injury/illness, any weight loss and reduction in BMI is associated with a decrease risk of injury/illness and delay in time to injury/illness. Our findings suggest that the decrease risk of injury/illness is incremental with greater reductions in the risk of injury/illness with increasing weight loss and the corresponding decreasing BMI. It should be emphasized that these health benefits were observed even among overweight and obese adults that lost weight but did not achieve a BMI below 25 kg/m^2^.

Based on prospective data, these longitudinal findings extend previous cross-sectional studies of US households that found self-reported injuries [22] and medically treated injuries [2] increased with increasing BMI. Participants with higher baseline BMIs were also injured earlier than were participants with lower baseline BMIs. Hootman et.al. observed this association between BMI and time to first injury in women (n = 609) but not in men (n = 2481) who regularly engaged in running, jogging, or walking for exercise: the women incurred an 8% increased risk of lower extremity injury for each unit increase in BMI [23]. Collectively, these studies suggest a linear dose-response relationship between BMI and injuries. In other words, any weight loss and reduction in BMI is associated with a decrease risk of injury/illness and delay in time to injury/illness.

In the present study, the dose-response relationships between BMI and injuries were not modified by the randomized exercise interventions: activity related injuries occurred among sedentary controls as well as in groups who were prescribed moderate-intensity exercise. This finding differs from previous cross-sectional studies which found that physical activity modified the dose-response relationship between BMI and injuries by reducing risk of injuries among overweight and obese adults [2,22]. In the present study, it is unlikely that the increase risk of injury or illness with BMI can be attributed to prior exercise experience; participants were sedentary at baseline, excluded if predisposed to exercise related injuries or reported bone or joint problems at screening, and randomized to the prescribed exercise intervention in the present study.

Although no association was observed between exercise and injury/illness, participants randomized to the exercise intervention did experience injuries that affected their ability to exercise. Incorporation of strengthening and/or flexibility exercises for the knees and back into an exercise program that emphasizes brisk walking may delay or prevent the most commonly reported injuries (lower-body musculoskeletal and back injuries/pain). Our findings also suggest that recovery from an injury/illness may be shorter for an overweight or obese adult randomized to an exercise intervention than for a sedentary overweight or obese adult.

In the present study, African Americans tended to suffer fewer injuries over time than whites. Although speculative, racial differences in body composition may explain this finding. Greater bone mineral density[24,25] and skeletal muscle mass[25] of African Americans compared to whites may be protective against injuries. African Americans have also been shown to have lower adiposity than whites for the same BMI [26]. If adiposity increases the risk of injury then whites would exhibit a higher risk of injury than African Americans as observed in this study. Additional research is warranted not only to confirm our findings but also to investigate the possible mechanisms of racial differences in injuries for overweight and obese adults.

There are several limitations with respect to data collection in this study. First, injury and illness data were collected at six month intervals instead of on an ongoing basis. Underreporting of injuries/illnesses due to recall bias may have occurred. Recent injuries/illnesses may be more readily remembered than those occurring further in the past; more severe injuries or those of longer duration might be better reported than minor injuries or those of short duration. Second, if a participant listed more than one injury or illness, we were not able to distinguish the impact of exercise on each injury. For example, a participant reported two injuries and indicated to what extent the injuries/illnesses were caused by exercise (not at all, a little, moderately, quite a bit, entirely). If the participant checked "moderately," that category would be applied to both injuries even if one was "not at all" caused by exercise (flu/cold/respiratory). This misclassification may explain why allergies, cold/flu/respiratory illness, and GI conditions were attributed to exercise (Table 3); Table 3 may thus underestimate specific injuries/illnesses not attributed to exercise and overestimate specific injuries/illnesses attributed to exercise. This misclassification may also have occurred in the lower body musculoskeletal injury but not in the any injury/illness analyses. Similar to the cause of injury, only total recovery time for all the injuries was available and not for each injury reported.

It should also be noted that the sedentary control group did receive a minimal intervention that encouraged exercise and healthy habits. During the 18-month intervention, the sedentary control group increased its leisure-time physical activity and cardiorespiratory fitness but the changes were less than those observed in the exercise intervention groups. This increase in physical activity or fitness levels in the control group may have biased the exercise results toward the null hypothesis.

Despite these limitations, the present study is the first to report the frequency and nature of injuries in a relatively large sample of overweight or obese adults enrolled in a weight loss or weight gain prevention study that emphasized walking for exercise. Enrollment of overweight as well as obese participants allowed the investigation of the influence of BMI on injuries/illnesses. In addition, the inclusion of a control group that did not receive a prescribed exercise intervention permitted the investigation of the causal relationship between exercise and injuries/illnesses.

## Conclusions

Consistently, regular exercise has been a component of successful long-term weight loss [17]. Although injuries are one of the perceived barriers to physical activity in individuals who never walk[27] and one of the primary reasons for exercise relapse[28], this study demonstrated a relatively low injury rate (1.8% per month) among overweight and obese individuals enrolled in an intervention program that emphasized a progressive increase in moderate intensity exercise. While there was no evidence that the exercise per se contributed to injury/illness, BMI contributed to the reported onset of first injury/illness in overweight and obese adults. The pattern of injuries/illnesses over time exhibited a linear dose-response relationship with more injuries/illnesses reported among adults with greater BMI. Any weight loss and corresponding reduction in BMI was found to be associated with a decrease risk of injury/illness and delay in time to injury/illness. Further research is warranted to determine if 1) weight loss in sedentary overweight and obese adults prior to initiating a moderate intensity exercise program reduces the risk of injury/illness compared to those who initiate a weight loss and exercise program simultaneously, 2) sedentary overweight and obese adults prone to injury due to exercise can reduce their risk of injury/illness by reducing their BMI prior to initiating and maintaining a moderate intensity exercise program, and 3) risk of injury/illness and exercise differs between obese adults with BMIs above 39.9 kg/m^2 ^and overweight and obese adults with BMI's between 25 and 39.9 kg/m^2 ^as reported in the present study.

## Abbreviations

BMI: Body Mass Index; OR: Odds Ratio; CI: Confidence Interval; SE: Standard Error; β: Beta coefficient.

## Competing interests

JJ is a scientific advisor for BodyMedia, Inc., Pittsburgh, PA and has a research grant funded by Beverage Institute for Health and Wellness. The authors have no other competing interests.

## Authors' contributions

CAJ conceived the current study, performed the statistical analysis, and wrote the manuscript. JMJ designed the larger clinical trials, acquired the data, participated in study discussions, and helped to revise the manuscript. The authors read and approved the final manuscript.

## References

[B1] Wadden T Stunkard A Handbook of Obesity Treatment 2002 New York: Guilford Press;

[B2] Finkelstein EA Chong H Prabhu M Trogdon JG Corso PS The Relationship between obesity and injuries among U.S. adults American Journal of Health Promotion 2007 21 460 468 17515011 10.4278/0890-1171-21.5.460

[B3] Nieman DC Nehlsen-Cannarella SL Henson DA Koch AJ Butterworth DE Fagoaga OR Utter A Immune response to exercise training and/or energy restriction in obese women Medicine & Science in Sports & Exercise 1998 30 679 686 10.1097/00005768-199805000-00006 9588608

[B4] Nieman DC Henson DA Nehlsen-Cannarella SL Ekkens M Utter AC Butterworth DE Fagoaga OR Influence of obesity on immune function Journal of the American Dietetic Association 1999 99 294 299 10.1016/S0002-8223(99)00077-2 10076580

[B5] Kesaniemi YK Danforth E Jr Jensen MD Kopelman PG Lefebvre P Reeder BA Dose-response issues concerning physical activity and health: an evidence-based symposium Medicine & Science in Sports & Exercise 2001 33 S351 358 10.1097/00005768-200106001-00003 11427759

[B6] Haskell WL Lee IM Pate RR Powell KE Blair SN Franklin BA Macera CAH Heath GW Thompson PD Bauman A Physical activity and public health: updated recommendation for adults from the American College of Sports Medicine and the American Heart Association Medicine & Science in Sports & Exercise 2007 39 1423 1434 10.1249/mss.0b013e3180616b27 17762377

[B7] Hootman JM Macera CA Ainsworth BE Addy CL Martin M Blair SN Epidemiology of musculoskeletal injuries among sedentary and physically active adults Medicine & Science in Sports & Exercise 2002 34 838 844 10.1097/00005768-200205000-00017 11984303

[B8] Matthews CE Ockene IS Freedson PS Rosal MC Merriam PA Hebert JR Moderate to vigorous physical activity and risk of upper-respiratory tract infection Medicine & Science in Sports & Exercise 2002 34 1242 1248 10.1097/00005768-200208000-00003 12165677

[B9] Nieman DC Is infection risk linked to exercise workload? Medicine & Science in Sports & Exercise 2000 32 S406 411 10.1097/00005768-200007001-00005 10910297

[B10] Nieman DC Henson DA Gusewitch G Physical activity and immune function in elderly women Medicine & Science in Sports & Exercise 1993 25 823 831 10.1249/00005768-199307000-00011 8350705

[B11] Nieman DC Nehlsen-Cannarella SI Henson DA Butterworth DE Fagoaga OR Warren BJ Rainwater MK Immune response to obesity and moderate weight loss International Journal of Obesity & Related Metabolic Disorders: Journal of the International Association for the Study of Obesity 1996 20 353 360 8680463

[B12] Scanga CB Verde TJ Paolone AM Anderson RE Wadden TA Effects of weight loss and exercise training on natural killer cell activity in obese women Medicine & Science in Sports & Exercise 1995 S68 10.1097/00005768-199812000-00002 9861597

[B13] Whaley M (Ed) ACSM's guidelines for exercise testing and prescription 2006 Philadelphia, PA:Lippincott Williams & Wilkins

[B14] Paffenbarger RS Jr Wing AL Hyde RT Physical activity as an index of heart attack risk in college alumni American Journal of Epidemiology 1978 108 161 175 707484 10.1093/oxfordjournals.aje.a112608

[B15] Kalbfleisch JD Prentice RL Hoboken NJ The Statistical Analysis of Failure Time Data 2002 Second John Wiley & Sons, Inc

[B16] Fitzmaurice G Laird N Ware J Applied Longitudinal Analysis 2004 Hoboken, New Jersey: John Wiley & Sons

[B17] Pronk NP Wing RR Physical activity and long-term maintenance of weight loss Obesity Research 1994 2 587 599 16358405 10.1002/j.1550-8528.1994.tb00110.x

[B18] Saris WH Blair SN van Baak MA Eaton SB Davies PS Di Pietro L Fogelholm M Rissanen A Schoeller D Swinburn B Tremblay A Westerterp KR Wyatt H How much physical activity is enough to prevent unhealthy weight gain? Outcome of the IASO 1st Stock Conference and consensus statement Obesity Reviews 2003 4 101 114 10.1046/j.1467-789X.2003.00101.x 12760445

[B19] 'Life Study Investigators' Pahor M Blair SN Espeland M Fielding R Gill TM Guralnik JM Hadley EC King AC Kritchevsky SB Maraldi C Miller ME Newman AB Rejeski WJ Romashkan S Studenski S Effects of a physical activity intervention on measures of physical performance: Results of the lifestyle interventions and independence for Elders Pilot (LIFE-P) study Journals of Gerontology Series A-Biological Sciences & Medical Sciences 2006 61 1157 1165 10.1093/gerona/61.11.1157 17167156

[B20] Goodrich D Larkin A Lowery J Holleman R Richardson C Adverse events among high-risk participants in a home-based walking study: a descriptive study International Journal of Behavioral Nutrition and Physical Activity 2007 4 20 10.1186/1479-5868-4-20 17521443 PMC1891313

[B21] Jeffery RW Wing RR Sherwood NE Tate DF Physical activity and weight loss: does prescribing higher physical activity goals improve outcome? American Journal of Clinical Nutrition 2003 78 684 689 14522725 10.1093/ajcn/78.4.684

[B22] Carlson S Hootman J Powell K Macera CA Heath GW Gilchrist J Kimsey C Jr Kohl H Self-reported injury and physical activity levels: United States 2000 to 2002 Ann Epidemiol 2006 1 8 10.1016/j.annepidem.2006.01.002 16626971

[B23] Hootman JM Macera CA Ainsworth BE Martin M Addy CL Blair SN Predictors of lower extremity injury among recreationally active adults Clinical Journal of Sport Medicine 2002 12 99 106 10.1097/00042752-200203000-00006 11953556

[B24] Jakicic JM Wing RR Differences in resting energy expenditure in African-American vs Caucasian overweight females International Journal of Obesity & Related Metabolic Disorders: Journal of the International Association for the Study of Obesity 1998 22 236 242 10.1038/sj.ijo.0800575 9539192

[B25] Wagner DR Heyward VH Measures of body composition in blacks and whites: a comparative review American Journal of Clinical Nutrition 2000 71 1392 1402 10837277 10.1093/ajcn/71.6.1392

[B26] Kleerekoper M Nelson DA Peterson EL Wilson PS Jacobsen G Longcope C Body composition and gonadal steroids in older white and black women Journal of Clinical Endocrinology & Metabolism 1994 79 775 779 10.1210/jcem.79.3.8077360 8077360

[B27] Eyler AA Brownson RC Bacak SJ Housemann RA The epidemiology of walking for physical activity in the United States Medicine & Science in Sports & Exercise 2003 35 1529 1536 10.1249/01.MSS.0000084622.39122.0C 12972873

[B28] Sallis JF Hovell MF Hofstetter CR Elder JP Faucher P Spry VM Barrington E Hackley M Lifetime history of relapse from exercise Addictive Behaviors 1990 15 573 579 10.1016/0306-4603(90)90059-7 2075855

